# Predictors of neonatal mortality among neonates admitted to the neonatal intensive care unit at Hawassa University Comprehensive Specialized Hospital, Sidama regional state, Ethiopia

**DOI:** 10.1186/s12887-024-04689-z

**Published:** 2024-04-03

**Authors:** Kefyalew Taye, Yenew Kebede, Desalegn Tsegaw, Worku Ketema

**Affiliations:** 1 Department of Pediatrics and Child Health, College of Medicine and Health Sciences, School of Medicine, Hawassa University, P.O.Box 1560, Hawassa, Ethiopia; 2 CEO at Makira Pediatrics and Child Health Specialty clinic, Hawassa, Sidama Ethiopia; 3 Department of Pediatrics and Child Health, College of Medicine and Health Sciences, School of Medicine, Debre Tabor University, Debre Tabor, Ethiopia; 4 College of Medicine and Health Sciences, School of Public Health, Hawassa University, P.O.Box 1560, Hawassa, Ethiopia

**Keywords:** Neonates, Neonatal intensive care unit, Neonatal mortality, Predictors

## Abstract

**Background:**

Despite promising efforts, substantial deaths occurred during the neonatal period. According to estimates from the World Health Organization (WHO), Ethiopia is among the top 10 nations with the highest number of neonatal deaths in 2020 alone. This staggering amount makes it difficult to achieve the SDG (Sustainable Development Goals) target that calls for all nations to work hard to meet a neonatal mortality rate target of ≤ 12 deaths per 1,000 live births by 2030. We evaluated neonatal mortality and it’s contributing factors among newborns admitted to the Neonatal Intensive Care Unit (NICU) at Hawassa University Comprehensive Specialized Hospital (HUCSH).

**Methods:**

A hospital-based retrospective cross-sectional study on neonates admitted to the NICU from May 2021 to April 2022 was carried out at Hawassa University Comprehensive Specialized Hospital. From the admitted 1044 cases over the study period, 225 babies were sampled using a systematic random sampling procedure. The relationship between variables was determined using bivariate and multivariable analyses, and statistically significant relations were indicated at p-values less than 0.05.

**Results:**

The magnitude of neonatal death was 14.2% (95% CI: 0.099–0.195). The most common causes of neonatal death were prematurity 14 (43.8%), sepsis 9 (28.1%), Perinatal asphyxia 6 (18.8%), and congenital malformations 3 (9.4%). The overall neonatal mortality rate was 28 per 1000 neonate days. Neonates who had birth asphyxia were 7.28 times more probable (AOR = 7.28; 95% CI: 2.367, 9.02) to die. Newborns who encountered infection within the NICU were 8.17 times more likely (AOR = 8.17; 95% CI: 1.84, 36.23) to die.

**Conclusion:**

The prevalence of newborn death is excessively high. The most common causes of mortality identified were prematurity, sepsis, perinatal asphyxia and congenital anomalies. To avert these causes, we demand that antenatal care services be implemented appropriately, delivery care quality be improved, and appropriate neonatal care and treatment be made available.

## Introduction

The neonatal mortality rate (NMR), which is the number of neonatal deaths per 1,000 live births in a given year, is a crucial sign of population health [1–5]. Around the moment of delivery, there is a heightened risk of mortality and severe morbidity. Perinatal mortality is the term used to describe the deaths of babies that occur between the weeks of gestation 28 and birth, including stillbirths and early neonatal deaths [2].

The first 28 days of life, or the neonatal phase, are a very vulnerable time for the baby as they complete several physiological changes needed for extrauterine existence [6, 7]. The neonatal intensive care unit (NICU), which uses advanced equipment and skilled staff to effectively offer specialized care for neonates, admits newborns who require immediate medical attention [8]. The result of the intricate interactions between neonatal, maternal, and healthcare-related factors is neonatal mortality. Neonatal mortality can be decreased by recognizing and comprehending aspects such as prenatal care, birth methods, and labor. Neonatal mortality continues to play a significant role despite the fact that significant efforts have been made and improvements in the under-five mortality rate have been observed. Neonatal deaths made up around half of the 5.3 million deaths of children under five worldwide [9]. The target 3.2 of the Sustainable Development Goals (SDGs) of reducing newborn mortality to at least 12 per 1,000 live births must be achieved in just around seven years [10].

According to the Ethiopian Mini-Demographic Health Survey (Mini-EDHS) study, newborn mortality is likely to increase, rising from 29 per 1,000 live births in 2016 to 33 per 1,000 live births in 2019 [11]. Notably, newborn deaths account for 56% of the under-five mortality rate in Ethiopia [11]. If maximum wide-ranging implementation tactics are not used to reduce neonatal death, Ethiopia risks missing SDG target 3.2. In order to prevent neonatal deaths and track the effectiveness of public health initiatives, it is crucial to establish the cause and contributing factors of mortality.

Knowing the disease patterns and causes in the Neonatal Intensive Care Unit (NICU), as well as the disease-specific mortality rate, can help guide the necessary efforts to reduce morbidity and mortality. There are no published documents that address this topic to our knowledge, so the goal of this study was to document the burden of neonatal death and to identify the predictors of neonatal death among admitted newborns at Hawassa University Comprehensive Specialized Hospital’s NICU (HUCSH).

## Method and materials

### Study setting

This study was aimed at assessing neonatal deaths and evaluating predictors of death in neonates admitted to the NICU of HUCSH. The study area was Hawassa City, which was located in the Sidama region on the shore of Lake Hawassa in the Great Rift Valley, 273 km south of Addis Ababa. Our research has been conducted at Hawassa University’s comprehensive specialized hospital, which was established 16 years ago by the Regional Health Bureau. The hospital was intended to serve the 3.5–5 million total population at the beginning and now serves the whole Sidama region, Southern Nations Nationalities and Peoples Region (SNNPR), and part of the Oromia region. HUCSH has a total of seven departments and fourteen units. Pediatrics and child health is one of the departments, where around 15,000 pediatric patients are treated per year.

The HUCSH NICU, inaugurated in 2014, has 35 beds equipped with advanced technologies for neonatal care. It is supported by multidisciplinary care and provides level III neonatal care. There are a total of 28 nurses assigned to the NICU. A consultant neonatologist, trained nurses, and pediatricians, together with pediatric residents and medical interns, provide services 24/7. It has four main classes: preterm, term, Kangaroo Mother Care (KMC), and backside. It is located near the hospital’s obstetric ward to receive high-risk newborns from this ward as quickly as possible. Apart from the hospital’s obstetric ward, the unit also receives neonates referred from other health facilities and homes.

The unit has 14 radiant warmers, 4 Continuous Positive Airway Pressure (CPAP), phototherapy, and oxygen concentrator machines. The unit provides outpatient and inpatient services, with estimated admissions of 100–150 newborns per month for inborn neonates as well as referred babies from surrounding provinces. Annual admissions at the NICU during the study period were 1044.

### Study design and period

A facility-based retrospective cross-sectional study of chart review was performed for the assessment of neonatal death ,and searching for predictors of death at HURH NICU over the past year prior to the study period(May 2021 to the end of April 2022), and the study period was from May 2022 to August 2022.

### Source population

All neonates who were admitted to NICU of HUCSH, Hawassa, Ethiopia during the study period.

### Study population

All systematically selected neonates that fulfilled the inclusion criteria who were admitted to the NICU of HUCSH, Hawassa, Ethiopia during the study period.

### Inclusion and Exclusion criteria

#### Inclusion criteria

All neonates who were admitted in the NICU of HUCSH and registered as died or alive within the first 28 days during the study period.

#### Exclusion criteria

- Medical records with incomplete information for the outcome variables.

### Study variables

#### Dependent variables

- Neonatal mortality- which was dichotomized as 1 if neonates had died and 0 if not.

#### Independent variables

1. Socio-demographic characteristics of the mother and the newborn

- Maternal
  - Age
  - Residence
  - Ethnicity
  - Occupation and income
  - ANC follow up

- Neonatal
  - Sex
  - Age

2. Obstetric factors

- Birth weight
- Mode of delivery
- Gestational age
- Apgar/immediate crying
- Resuscitation at delivery
- Duration of labor
- Duration of rupture of the membrane
- Maternal fever
- Parity
- Prior pregnancy loss

3. Neonatal inpatient conditions

- Evidence of sepsis
- Length of stay before discharge (alive/death)
- Time during death date

### Operational definitions [12–14]

Neonatal mortality is the death of the newborn during the first 28 completed days after live birth (days 0–27) after admission to the NICU and before discharge, as confirmed and recorded on the chart.

Early neonatal death is a death during the first 7 completed days after live birth (days 0–6).

Antenatal care visit: any history of visit or follow-up during the current or index pregnancy at any health institution for a checkup of pregnancy and designated or recorded on a chart.

Intrapartum complications are complications that occur after the onset of labor, including intrapartum bleeding, obstructed labor, prolonged labor, eclampsia, chorioamnionitis, and others.

Congenital malformation is a body deformity or deformities, structural or functional anomalies, that occur during intrauterine life and can be identified prenatally, at birth, or sometimes only later in infancy, and that is believed to have an impact on the health of the baby. It is diagnosed and recorded on charts by professionals on admission.

Hypoglycemia is a measure of low blood glucose (40 mg/dl) that was diagnosed and recorded on charts by professionals on admission.

Hypothermia is a low-body temperature measurement (< 36.5 °C), diagnosed and recorded on or during admission of neonates.

Birth asphyxia is diagnosed whenever a neonate has an APGAR score of 6 in the 5th minute and/or if he or she does not cry immediately after birth, has respiratory distress, floppiness, loss of consciousness, the presence of convulsions, and a loss of neonatal reflexes.

Birth weight is classified using the WHO weight classification: very low birth weight is any child with a birth weight of 1,500 g, while low birth weight is any child with a birth weight of 2,500 g.

Premature rupture of membranes (PROM) is the spontaneous leakage of amniotic fluid from the amniotic sac, occurring after 28 weeks of gestation and before the onset of labor. It was diagnosed and recorded on charts by professionals on admission.

Prolonged rupture of membranes (PROM) is considered when the duration of the leakage is more than 18 h before delivery.

### Sample size determination and procedures

The sample size was calculated by using a single population proportion formula with assumptions of a confidence level of 95% = 1.96, a margin of error (d) = 0.05, and the magnitude of neonatal death (*p* = 0.23) from a previous study conducted at the NICU of Gondar Referral Hospital [15].Considering a 10% incompleteness rate, the final sample size was 235.

The study participants were selected by systematic random sampling using the registration numbers of the neonates. Among a total of 1044 neonatal registration numbers, every fifth participant was selected. The first study unit was selected using the lottery method. Estimation of neonatal mortality rate (NMR): The median follow-up period of selected risky neonates for the study was determined. The product of the number of days of the median follow-up period and the total number of neonates followed gives neonate-days. The overall incidence of neonatal mortality was calculated as a ratio of neonatal deaths to neonate days per 1000 neonate days.

### Data collection and materials

A data extraction checklist that was modified and adjusted from various literary works was used to review the medical records and collect the data [15, 16]. The following variables were included in the data using the data collection tool: the clinical diagnosis at admission, sex, maternal and neonatal age, complications during pregnancy, gestational age, antenatal care visits, birth weight, place of birth (HUSCH, other, or home), the neonate’s body temperature, random blood sugar at admission, the mode of delivery, the outcome (survival or death) at discharge, and the length of stay prior to discharge. The International Classification of Diseases in its tenth iteration (International Statistical Classification of Diseases and Related Health Problems, ICD-10-WHO, version 2015) was used to classify clinical diagnoses (congenital deformity, prematurity, birth asphyxia, infection).

### Data quality assurance

A data-gathering tool that was properly designed and structured was used. During data collection and data input, data collectors were trained and strictly supervised. The data collectors were medical interns and residents undergoing training to improve their comprehension and interpretation of patient medical charts. The lead investigator cross-checked 10% of the gathered data with medical records and ensured its consistency after data collection.

### Data management and analysis

At the end of each day, the data were reviewed for completeness and consistency. It was cleaned, modified, programmed, and entered into EpiData software version 4.6. The data were then exported into SPSS 25 for statistical analysis. During the analysis, the frequency distribution and percentage of various variables were computed to describe and summarize the respondents’ basic socio-demographic characteristics. To provide an overview of the variables, the findings were presented in the form of frequency tables, pie charts, and graphs. Neonatal survival (lived or died within the neonatal period of 28 days) was defined as the binary outcome for bivariate and multivariate studies examining risk factors. Binary regression analysis was used to first look into the bivariate relationship between each independent variable and the result. To reduce the impact of confounding variables and pinpoint the primary causes of inpatient neonatal death, the variables with a p-value of 0.20 during the bivariate analysis were used as candidate variables for a multivariate logistic regression analysis model. Neonatal mortality was determined using an adjusted odds ratio (AOR) at a 95% confidence interval (CI) to demonstrate the strength of the link, and statistical significance was deemed to exist at a p-value of 0.05. The Chi square and Fisher’s exact tests were used to determine the statistical significance of the differences between the groups. The median follow-up time for the study’s risky neonates was established. Neonatal days are calculated as the median follow-up period divided by the total number of neonates followed. The ratio of neonatal fatalities to neonatal days per 1,000 neonatal days, which represents the total incidence of neonatal mortality.

## Results

During the most recent one-year study period, a total of 1044 neonates admitted to the NICU were registered in the HIMS registry book. Of these, 225 neonates’ charts were reviewed and included in the study. Out of the 235 newborns who were chosen, 10 had medical records that were incomplete; therefore, 225 cases were chosen with an overall review rate of 96%. In the 225-person sampled retrospective survey study group, 127 (56.4%) of the babies were boys born to mothers who were between the ages of 18 and 45. The median age of the neonatal mothers, who made up 84% of the population, was 26 years old. Within the first 24 h of life, 154 neonates (68.4%) were admitted, and between one and seven days, 42 newborns (18.7%) were admitted, making up roughly two thirds of the total. Additionally, with a median of 5.0 days and an interquartile range of 9 days, the average length of stay in neonatal critical care units was 8.1 days. (Table 1).

**Table 1:** Socio-demographic characteristics of neonates and mothers of the neonates admitted at the NICU of the HUCSH, Southern Ethiopia, 2022

Variable	Categories	Neonatal Outcome		*p*-value
		Death	Survival	
Maternal age in years	< 20	1 (9.1%)	10 (90.9%)	0.317
	20–34	25 (13.2%)	164 (86.8%)	Reference
	≥ 35	6 (24.0%)	19 (76.0%)	0.157
Sex of Neonates	Female	15 (15.3%)	83 (84.7%)	Reference
	Male	17 (13.4%)	110 (86.6%)	0.683
Age of neonates at admission in day/s	< 1	25 (16.2%)	129 (83.8%)	Reference
	1–6	5 (11.9%)	37 (88.1%)	0.209
	≥ 7	2 (6.9%)	27 (93.1%)	0.492

### Maternal Obstetrics Characteristics

About a quarter, 54 (24%) of mothers of neonates, had suboptimal, less than four Antenatal Care (ANC), follow-up visits before the delivery of the current newborns. Ten (4.4%) of the mothers had no ANC follow-up. There were 8 (3.6%) mothers who gave birth at their homes. The majority, 204 (90.7%) of mothers, had singleton deliveries. In this study, ninety-eight (43.6%) of mothers were primiparous. Sixty-three (28%) of the mothers had delivered for the second time; a single mother (0.9) was found to have a total of nine delivery histories. One-fifth, or 46 (20.4%), of mothers of neonates encountered index pregnancy complications like preeclampsia (16), chorioamnionitis (8), antepartum hemorrhage (7), eclampsia (5), and others (diabetes mellitus, obstructed labor, poly/oligohydramnios) (10). Thirty-eight (17.5%) of the mothers faced intraprtum complications like prolonged rupture of the membrane (PROM) before the onset of labor, which occurred in 20 (8.9%) of the mothers, while non-reassuring heart rate pattern (NRHRP) with bradycardia or tachycardia secondary to meconium-stained amniotic fluid was documented in 12 (5.3%) of the parturient mothers. The proportion of neonates directly transferred from the obstetric ward of HUCSH was 71.6% (161), greater than referred cases, 28.4% (64).) (Table 2).

**Table 2:** Maternal obstetric characteristics of neonates admitted to the NICU of the HUCSH, Southern Ethiopia, 2022

Variable	Categories	Neonatal Outcome		*p*-value
		Death	Survival	
Number of ANC	≥ 4 visits	19(13.2%)	152(13.2%)	Reference
	1–3 visits	9 (20.5%)	35(79.5%)	0.015
	No single visit	4 (40.0%)	6 (60.0%)	0.106
Place of delivery	Health institution	30 (13.8%)	187 (86.2%)	Reference
	Home	2 (25.0%)	6 (75.0%)	0.384
Type of pregnancy	Singleton	28 (13.7%)	176 (86.3%)	Reference
	Multiple	4 (19.0%)	17 (81.0%)	0.508
Parity	< 2	12 (12.2%)	86 (87.8%)	Reference
	2–4	15 (13.6%)	95 (86.4%)	0.766
	> 4	5 (29.4%)	12 (70.6%)	0.075
Index pregnancy complications	No	24(13.4%)	155 (88.6%)	Reference
	Yes	8 (17.4%)	38 (82.6%)	0.491
Intrapartum complications	No	23 (12.8%)	156 (87.2%)	Reference
	Yes	9 (23.7%)	29 (76.3%)	0.092
Duration of PROM	< 18 h	27 (13.2%)	178 (86.8%)	Reference
	≥ 18 h	5 (25.0% )	15 (75.0%)	0.157
Admission status	Transferred	24 (14.9%)	137 (85.1%)	Reference
	Referred in	8 (12.5%)	56 (87.5%)	0.641

### Description of Neonatal related characteristics

About a quarter of the newborns, 55 (24.4%), were less than 37 weeks of gestational age. One neonate (0.4%) was born post term. However, 59 (26.2%), 14 (6.2%), and 2 (0.9%) of the hospitalized neonates were LBW, VLBW, and ELBW, respectively. Regarding the major vital signs of neonates at admission, the respiratory rate was normal for 119 (52%) neonates; approximately one-third, 68 (30.2) and 43 (19.1%), were hypothermic and febrile, respectively. Hypoglycemia was found in 14 (6.2%) of the infants. Perinatal asphyxia was observed in 25 (11.1%) of the neonates, as shown by either failure to cry shortly after birth, necessitating resuscitation, a low Apgar score, or both. 122 (54.2%) of the neonates were admitted with the diagnosis of early or late-onset sepsis or infection inside the NICU setting. Hospital-acquired infection (HAI) was found in 11 (4.9%) of the newborns who died in the NICU. The 37 (16.4%) newborns were found to have a variety of congenital abnormalities. Meningomyelocele (MMC) was the commonest (12, 5.5%) malformation, followed by anorectal malformations (3), Hirschsprung’s disease (3), small intestinal atresia (3), congenital heart disease (2), choanal atresia (2), bladder extrophy (2), cleft lip and palate (2), and others (tracheoesophageal fistula, hypospadias, encephalocele, Aqueductal stenosis, Edward syndrome, Down syndrome) (1 each). After admission, fifty eight (25.7%) neonates either presented with or developed neonatal hyperbilirubinemia or jaundice. (Table 3).

**Table 3:** Neonatal-related characteristics of neonates admitted to the neonate intensive care unit (NICU) of the HUCSH, Southern Ethiopia, 2022

Variable	Categories	Neonatal Outcome		*p*-value
		Death	Survival	
GA (wks)	≥ 37	12 (7.1%)	158 (92.9%)	Reference
	< 37	20(36.4%)	35 (63.6%)	0.001
Birth (g)	2500–3999	9 (6.4%)	131 (93.6%)	Reference
	< 2500	23 (30.7%)	52 (69.3%)	0.001
Respiratory rate ( breath/min)	31–60	13 (10.9%)	106 (89.1%)	Reference
	≥ 61	16 (15.7%)	86 (84.3%)	0.019
	≤ 30	3 (75.0%)	1 (25.0%)	0.007
Body temperature(^O^C)	36.5–37.5	11 (9.6%)	103 (90.4%)	Reference
	≥ 37.6	4 (9.3%)	39 (90.7%)	0.048
	< 36.5	17 (25.0%)	51 (75.0%)	0.007
Hypoglycemia	No	27 (12.8%)	184 (87.2%)	Reference
	Yes	5 (35.7% )	9 (64.3%)	0.025
Perinatal asphyxia	No	24 (12.0%)	176 (88.0%)	Reference
	Yes	8 (32.0%)	17 (68.0%)	0.010
Sepsis	No	13 (12.6%)	90(87.4%)	Reference
	Yes	19 (15.6%)	103 (84.4%)	0.570
Hospital acquired infection	No	21 (10.9%)	172 (89.1%)	Reference
	Yes	11 (34.4%)	21 (65.6%)	0.001
Congenital malformations	No	20 (13.2%)	132 (86.8%)	Reference
	Yes	7 (18.9%)	30 (81.1%)	0.372
Jaundice	No	24 (14.4% )	143 (85.6%)	Reference
	Yes	8 (13.8%)	50 (86.2%)	0.914

#### Prevalence of Neonatal Mortality and Neonatal Mortality Rate( NMR)

The majority of the neonates admitted to the HUCSH NICU, 193 (85.8%), were discharged alive, while 32 (14.2%) died before discharge. As a result, the prevalence of newborn death was 14.2% (95% confidence interval: 0.099–0.195). The average hospital stay for the 225 hospitalized neonates was 5 days, accounting for 1,125 neonate-days in the study. Taking 32 neonatal fatalities over the follow-up period into account, the overall neonatal mortality rate was 28 per 1000 neonate days. The majority, 23 (71.9%) of neonates perished before their first seven days of life, and three (9.4%) died on the first day of life.

#### Immediate Causes of Neonatal Death

Prematurity (14, 43.8%), sepsis (9, 28.1%), prenatal asphyxia (6, 18.8%), and congenital abnormalities (3, 9.4%) were the most frequent causes of newborn death. Six (42.9%) of the newborns with sepsis were also diagnosed with prematurity as their direct cause of death. Despite birth asphyxia being listed as the initial cause of death, two (33.3%) of the neonates additionally developed sepsis. Additionally, sepsis was present in 2/3 (66.6%) of neonatal deaths that were attributed to congenital deformity as the direct cause of death. Two (14.3%) of the newborns who died from asphyxia were preterm.

### Factors Associated With Neonatal Mortality

Numerous factors, including the number of ANC visits, parity, intrapartum complications, premature rupture of the membranes (PROM), neonatal gestational age (GA), neonatal birth weight, hypothermia, hypoglycemia, perinatal asphyxia, and hospital-acquired infection (HAI), were taken into consideration for multivariate binary logistic regression analysis to control the effect of confounders of binary logistic regression. Multivariate logistic regression research revealed significant relationships between variables such as neonatal gestational age, birth weight, hypothermia, hypoglycemia, perinatal hypoxia, HAI, and neonatal death.

Neonatal mortality was more than 4 times more likely to occur in preterm neonates compared to those whose GA was at least 37 weeks (AOR = 4.21; 95% CI: 2.43, 8.69). Neonates with low birth weights were 5.12 times more likely to die from neonatal causes than infants with normal birth weights (AOR = 5.125; 95% CI: 1.56, 10.06). Neonatal mortality was 1.66 and 4.16 times more likely to happen in hypothermic and hypoglycemia neonates than in neonates without the conditions (AOR = 1.66; 95% CI: 1.56, 6.84) and correspondingly (AOR = 4.16; 95% CI: 1.80, 6.04). The odds of a neonate dying during their newborn period were 7.28 times higher for those who suffered birth asphyxia (AOR = 7.28; 95% CI: 2.367, 9.02) than for those who did not. When a newborn was infected in the NICU, neonatal mortality was 8.17 times more likely to occur (AOR = 8.17; 95% CI: 1.84, 36.23). (Table 4).

**Table 4:** Factors affecting neonatal mortality at the NICU of the HUCSH, Southern Ethiopia, 2022

Variable	Categories	Neonatal Outcome		COR with 95% CI	AOR with 95% CI		*p*-value
		Death	Survival				
Number of ANC visits	≥ 4	19 (13.2%)	152 (13.2%)	1		1	Ref.
	1–3	9 (20.5%)	35 (79.5%)	5.33 (1.38–20.62		2.36 (0.35–9.04)	0.381
	No	4 (40.0%)	6 (60.0%)	2.59 (0.60–9.19)		2.18 (0.27–17.53)	0.465
Parity	< 2	12 (12.2%)	86 (87.8%)	1		1	
	2–4	15 (13.6%)	95 (86.4%)	0.88 (0.39–1.99)		0.20 (0.42–11.28)	0.194
	> 4	5 (29.4%)	12 (70.6%)	0.34 (0.10–1.12)		0.13 (0.41–12.53)	0.125
Intrapartumcomplications	No	23 (12.8%)	156 (87.2%)	1		1	Ref.
	Yes	9 (23.7%)	29 (76.3%)	0.48 (0.20–1.13)		1.68 (0.40–7.05)	0.478
Duration of PROM	< 12 h	27 (13.2%)	178 (86.8%)	1		1	Ref.
	≥ 12 h	5 (25.0%)	15 (75.0%)	0.46 (0.15–1.35)		1.64 (0.29–9.31)	0.575
GA in weeks	≥ 37	12 (7.1%)	158 (92.9%)	1		1	Ref.
	< 37	20 (36.4%)	35 (63.6%)	7.52 (3.37–9.81)		4.21(2.43–8.69)	0.048^*^
Birth weight in grams	≥ 2500	9 (6.0%)	141 (94.0%)	1		1	Ref.
	< 2500	23 (30.7%)	52 (69.3%)	34.47 (9.84–12.74)		5.12 (4.01–12.34)	0.01*
Hypother mia	No	11 (9.6%)	103 (90.4%)	1		1	Ref.
	Yes	4 (9.3%)	39 (90.7%)	2.49 (1.15–5.38)		1.66 (1.56–6.84)	0.038^*^
Hypogly-cemia	No	27 (12.8%)	184 (87.2%)	1		1	Ref.
	Yes	5 (35.7% )	9 (64.3%)	3.78 (1.18–12.14)		4.16 (1.80–6.04)	0.03^*^
Perinatal asphyxia	No	24 (12.0%)	176 (88.0%)	1		1	Ref.
	Yes	8 (32.0%)	17 (68.0%)	3.45 (1.34–8.85)		7.28 (2.367–9.02)	0.001^**^
HAI	No	21 (10.9%)	172 (89.1%)	1		1	Ref.
	Yes	11 (34.4%)	21 (65.6%)	4.29 (1.82–10.13)		8.17 (1.84–36.23)	0.02^*^

P^*^< 0.05, p^*^< 0.01, COR- Crude odds ratio, AOR-Adjusted odds ratio, CI-Confidence interval

## Discussion

This study was done retrospectively on neonates admitted to NICU where intensive care is required to save the lives of newborns with critical clinical conditions and struggling to adapt to the new environment. In the developing world: poor quality of care during antenatal, intrapartum, and postpartum periods contributes most to neonatal mortality. Hence, continuous assessment is needed as neonatal mortality has not declined, rather, the mortality has continued to contribute a huge share (47%) of the overall under-5 mortality.

According to the current study, the overall neonatal death rate was 28 per 1000 neonate days, and the proportion of neonatal loss was 14.2% (95% CI: 0.099–0.195). Studies conducted in Hiwot Fana Specialized University Hospital, Eastern Ethiopia, at 14.3% infant mortality rates were found to be congruent with this 14.2% neonatal death magnitude [17];Jimma University Medical Center, Southwest Ethiopia at 13.3% [18] and Dire Dawa city, Ethiopia at11.4% [19].But the finding was lower compared with studies conducted in Ghana at 20.2% (17); Debre Markos, Northwest Ethiopia, at 21.3% (18); Mizan Tepi University Teaching Hospital, Southwest Ethiopia, at 22.8% (19); and Gondar Referral Hospital, Northwest Ethiopia, at 23.1% (13). However, the prevalence of neonatal death was much higher than studies conducted in the Somali Region, Ethiopia, at 5.7% (20); the North Gondar Zone, Ethiopia, at 4.4% (21); and the East Wollega Zone, Ethiopia, at 6.6% (22). The neonatal hospital mortality rate of 28 per 1000 neonates-days was highly consistent with a similar study done in Wolaita Sodo, Ethiopia, at 27 per 1000 neonates-days (23). However, the finding was higher than the mortality rate determined in Hadiya zone, southern Ethiopia, at 25 per 1000 neonates-days(24).The discrepancy might be due to differences in sample sizes, in sociocultural and socioeconomic aspects, in health service utilization, including giving birth at health institutions by skilled care providers and health seeking for sick neonates, the variation in health institution setup, and economic disparities among study participants (19, 25, 26). As per this study, which is in line with many others, neonates delivered before 37 weeks of gestational age (GA) were measured as having a statistically significant association with the episode of neonatal mortality that was over 4 times more likely (AOR = 4.21; 95% CI: 2.43, 8.69) when compared to neonatal mortality in neonates with GA of 37 weeks and above. According to a study conducted in Rwanda, newborns born at gestational age (GA) less than 37 weeks had a 3.1-times higher mortality rate than newborns born at GA of 37 weeks or higher [20]. In addition, a study in Kenya found that the risk of newborn death attributable to prematurity is more than 7.0 times higher when compared to controls with a GA of 37 weeks and above [21]. Preterm babies are more likely to die than mature neonates by 3.3 and 2.5 times, respectively, according to research done in Adama and Dilla, Ethiopia [22, 23]. Prematurity may lead to poor neonatal outcomes due to the immaturity of the respiratory and circulatory systems, susceptibility to infection, hypothermia, and failure to adjust to the external environment [24–27].

Newborns with low birth weights had a neonatal death rate that was 5.12 times higher than that of infants with normal birth weights (AOR = 5.125; 95% CI: 1.56, 10.06). According to reports, newborns with low birth weight (LBW) were 1.54 times more likely to die in hospitals than newborns with birth weights of 2500 g and higher. This relationship between low birth weight and neonatal mortality events was significant, according to the report of a study from Jimma, Ethiopia [18]; which is consistent with reports of studies from Adama, Ethiopia [22]and Dilla, Ethiopia [23] measuring 1.6 and 2.44 times more likely deaths to LBW neonates as compared to neonates with birth weight ≥ 2500 g. In keeping with a study from Wolaita, Ethiopia, neonates with low birth weights have a nine-fold higher risk of dying than those with normal birth weights [28]. Due to metabolic, hematologic, and immunological functions, low birth weight may increase the risk of infection and hypothermia [29, 30].

Neonatal death was 1.66 times more likely in hypothermic newborns (AOR = 1.66; 95% CI: 1.56, 684) compared to neonates who were not hypothermic. The results were similar to a study in Dire Dawa, Ethiopia, that found babies with hypothermia had a 2.5 likelihood of neonatal death [16]. The association between hypothermia and conditions like hypotension, hypoxia, hypoglycemia, bradycardia, disseminated intravascular coagulation, irregular and sluggish breathing, and shock is one reason that has been proposed [31], which, in turn, increase the likelihood of death.

Compared to neonates who were normoglycemic, neonates with neonatal hypoglycemia had an odds ratio of 4.16 (AOR = 4.16; 95% CI: 1.80, 6.04) for neonatal death. The prevalence is higher than the reported value from Macedonia, which was 2.38% [31]. The fact that hypoglycemia causes neurological impairment, bradycardia, irregular and slow breathing, and apnea may be an explanation for the substantial mortality rate in hypoglycemic neonates [32].

Birth asphyxia, defined as failing to breathe immediately after birth, requiring resuscitation after birth, or having a poor APGAR score, was associated with a 7.28 times greater likelihood (AOR = 7.28; 95%CI: 2.367, 9.02) of dying during the newborn period than those who were not asphyxiated. According to a study conducted in the Amhara area of Ethiopia, the prevalence of birth asphyxia was reported to be 22.6% [95% CI 19.2% − 26.4%] in the first minute of birth [33]; strongly linked with primipara, prolonged labor, and Prolonged membrane rupture. The prevalence of perinatal asphyxia in a report from Northwest Ethiopia (31) was 19.8% (95% CI: 15.9, 24.2), which was significantly associated with the absence of maternal formal education (AOR = 4.09, 95% CI: 1.25, 13.38), pregnancy-induced hypertension (AOR = 4.07, 95% CI: 1.76, 9.40), antepartum hemorrhage (AOR = 6.35, 95% CI: 1.68, 23.97), prolonged duration of labor (AOR = 3.69, 95% CI: 1.68, 8.10), instrumental delivery (AOR = 3.17, 95% CI: 2.19, 9.26), and meconium-stained amniotic fluid (AOR = 4.50, 95% CI: 2.19, 9.26). Neonatal sepsis is the main factor in neonatal morbidity and mortality on a global scale. According to this study, newborns who tested positive for a hospital-acquired illness were 8.17 times more likely to pass away than newborns who did not have HAI (AOR = 8.17, 95% CI: 1.84, 36.23). In various investigations, neonatal sepsis has been identified as a substantial risk factor for newborn mortality. The results of a study from Dilla, Ethiopia, showed that newborns with sepsis were 2.45 times more likely to die prematurely than neonates without sepsis [23]; which is similar to the report of a study in Adama, Ethiopia (19), which revealed neonates with sepsis were 2.4 times more likely to face death when compared to neonates who were not having sepsis. According to a report from a study conducted in Eastern Ethiopia, the total prevalence of sepsis was 45.8%, and it was strongly linked to things like prolonged membrane rupture, prelacteal feeding, and a low APGAR score [34].

### Limitations of the Study

A smaller sample size could reduce the representativeness of the source population, and the data was secondary, with the potential for incompleteness. The fact that we used glucose 40 mg/dl for defining hypoglycemia might have resulted in underdiagnoses especially on the first day of admission. Furthermore, because the study was cross-sectional, a cause-and-effect relationship might not be demonstrated.

## Conclusion

The prevalence of newborn death is excessively high. The most common causes of mortality identified were prematurity, sepsis, perinatal asphyxia and congenital anomalies. To avert these causes, and to remedy the disparities that have been identified, we demand that antenatal care services be implemented appropriately, delivery care quality be improved, and appropriate neonatal care and treatment be made available.

## Data Availability

The correspondent author will provide additional data of this article upon reasonable request.
